# First-Year Experience of Managing Urology Patients With Home Uroflowmetry: Descriptive Retrospective Analysis

**DOI:** 10.2196/51019

**Published:** 2023-10-17

**Authors:** Lola Bladt, Ardavan Kashtiara, Wouter Platteau, Stefan De Wachter, Gunter De Win

**Affiliations:** 1 Product Development Faculty of Design Sciences University of Antwerp Antwerp Belgium; 2 Antwerp Surgical Training, Anatomy and Research Centre Faculty of Medicine and Health Sciences University of Antwerp Antwerp Belgium; 3 Department of Urology University Hospital Antwerp Antwerp Belgium

**Keywords:** lower urinary tract symptoms, home uroflowmetry, automated bladder diary, homeflow, hospiflow, mobile phone

## Abstract

**Background:**

Lower urinary tract symptoms affect a large number of people of all ages and sexes. The clinical assessment typically involves a bladder diary and uroflowmetry test. Conventional paper-based diaries are affected by low patient compliance, whereas in-clinic uroflowmetry measurement face challenges such as patient stress and inconvenience factors. Home uroflowmetry and automated bladder diaries are believed to overcome these limitations.

**Objective:**

In this study, we present our first-year experience of managing urological patients using Minze homeflow, which combines home uroflowmetry and automated bladder diaries. Our objective was 2-fold: first, to provide a description of the reasons for using homeflow and second, to compare the data obtained from homeflow with the data obtained from in-clinic uroflowmetry (hospiflow).

**Methods:**

A descriptive retrospective analysis was conducted using Minze homeflow between July 2019 and July 2020 at a tertiary university hospital. The device comprises a Bluetooth-connected gravimetric uroflowmeter, a patient smartphone app, and a cloud-based clinician portal. Descriptive statistics, Bland-Altman plots, the McNemar test, and the Wilcoxon signed rank test were used for data analysis.

**Results:**

The device was offered to 166 patients, including 91 pediatric and 75 adult patients. In total, 3214 homeflows and 129 hospiflows were recorded. Homeflow proved valuable for diagnosis, particularly in cases where hospiflow was unreliable or unsuccessful, especially in young children. It confirmed or excluded abnormal hospiflow results and provided comprehensive data with multiple measurements taken at various bladder volumes, urge levels, and times of the day. As a result, we found that approximately one-fourth of the patients with abnormal flow curves in the clinic had normal bell-shaped flow curves at home. Furthermore, homeflow offers the advantage of providing an individual’s plot of maximum flow rate (Q-max) versus voided volume as well as an average or median result. Our findings revealed that a considerable percentage of patients (22/76, 29% for pediatric patients and 24/50, 48% for adult patients) had a Q-max measurement from hospiflow falling outside the range of homeflow measurements. This discrepancy may be attributed to the unnatural nature of the hospiflow test, resulting in nonrepresentative uroflow curves and an underestimation of Q-max, as confirmed by the Bland-Altman plot analysis. The mean difference for Q-max was −3.1 mL/s (with an upper limit of agreement of 13 mL/s and a lower limit of agreement of −19.2 mL/s), which was statistically significant (Wilcoxon signed rank test: V=2019.5; *P*<.001). Given its enhanced reliability, homeflow serves as a valuable tool not only for diagnosis but also for follow-up, allowing for the evaluation of treatment effectiveness and home monitoring of postoperative and recurrent interventions.

**Conclusions:**

Our first-year experience with Minze homeflow demonstrated its feasibility and usefulness in the diagnosis and follow-up of various patient categories. Homeflow provided more reliable and comprehensive voiding data compared with hospiflow.

## Introduction

### State-of-the-Art Assessment Methods for Lower Urinary Tract Symptoms

**Figure 1:**
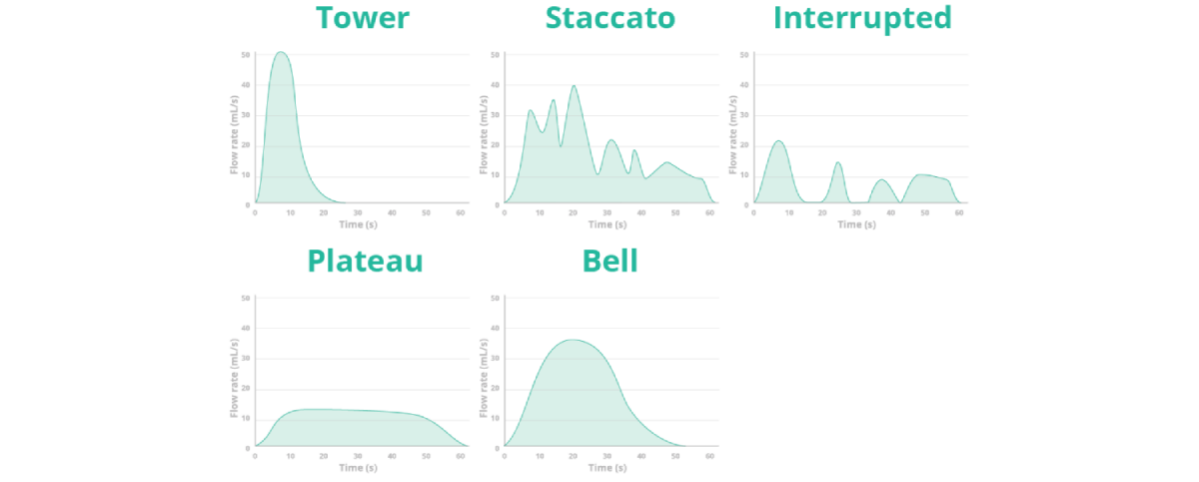
Type of uroflowmetry curve: tower, staccato, interrupted, plateau, and bell.

According to global estimates, over 2.3 billion people are affected by lower urinary tract symptoms (LUTS) across all sexes and all age groups [1]. LUTS encompass a group of symptoms related to problems in the bladder, urethra, pelvic floor, urinary sphincter, and prostate in men. It has a substantial impact on the quality of life, sexual function, and mental health. The incidence of LUTS increases with age, making it a significant public health concern [2]. A thorough evaluation of LUTS should include the assessment of medical history, a bladder diary, and a uroflowmetry assessment (with postvoid residual measurement) [3-6]. Uroflowmetry measures urinary flow during voiding and is suitable for both adults and toilet-trained children owing to its noninvasive nature. It provides a simple and cost-effective approach with a wide range of diagnostic information. Specifically, the uroflow curve holds considerable diagnostic value, with a tower-shaped curve potentially indicating an overactive bladder, a staccato curve associated with a pelvic floor dysfunction, an interrupted curve suggesting straining owing to an underactive bladder, plateau curves commonly seen in cases of anatomical obstruction of flow, and a bell-shaped curve generally considered normal [5] (Figure 1).

**Figure 2:**
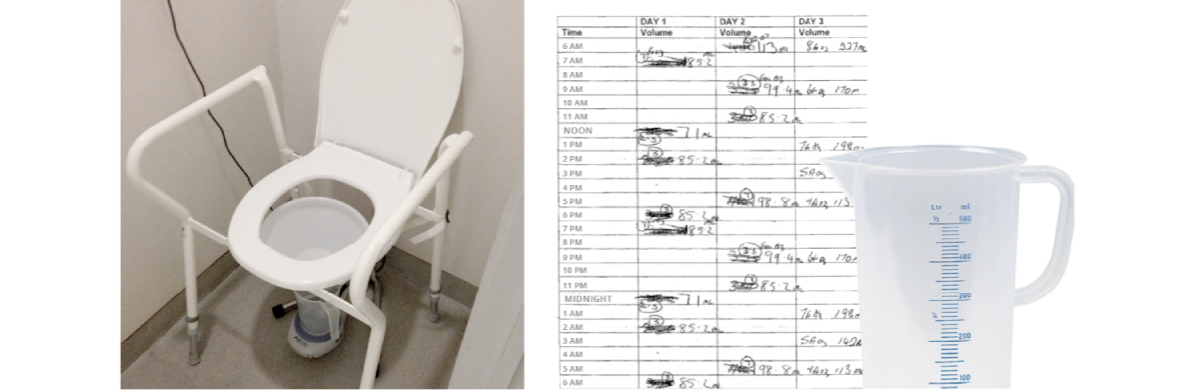
State-of-the-art lower urinary tract symptoms assessment methods: in-clinic uroflowmetry (left) and paper-based bladder diary (right).

In-clinic uroflowmetry (Figure 2) measurements may face inherent challenges, such as patient reluctance because of inconvenience factors such as travel, cost, and time [7]. Furthermore, psychological stress caused by the in-clinic examination can have a significant impact on test results [8-10], particularly in pediatric uroflowmetry [11,12]. When patients report that the test is not representative or when the results show abnormalities, it is recommended to repeat the test [4]. For pediatric patients, guidelines recommend conducting at least 2 uroflowmetry tests in any case and a third test if the first 2 are inconsistent [13,14]. Moreover, it is recommended to evaluate uroflow parameters with a minimum voided volume (V-void) of 150 mL in adults [15] and a minimum V-void of at least 50% of the age-expected bladder capacity (calculated as 30 + [age in years × 30] in mL) [16,17] for children aged up to 12 years [5]. Given the requirements and challenges associated with in-clinic uroflowmetry, it is believed that conducting the test at home may yield better results [18,19]. It allows for obtaining multiple natural flow traces and averaging the results for a more comprehensive understanding of the patient’s urinary function [20]. In addition, it may reduce psychological stress and attract more patients, while also eliminating time-related restrictions that may ultimately reduce the economic burden on health care institutions. However, to ensure reliable results that are suitable for diagnostic purposes, it is crucial to use a robust, mobile, user-friendly, and accurate home uroflowmeter device [7].

In addition to uroflowmetry, several international guidelines endorse the use of bladder diaries for the clinical evaluation of patients with LUTS [5,15,21-27]. They provide a standard method for assessing fluid intake, urine output, incontinence episodes, and bladder sensation (such as urgency) over 2 to 3 days. However, conventional paper-based bladder diaries (Figure 2) are often considered cumbersome and inconvenient by patients [28], resulting in low patient compliance. To overcome these limitations, various electronic bladder diaries have been developed [29-37]. It has been suggested that they facilitate a more effective and intuitive way of data entry, storage, and management compared with traditional paper-based diaries [33]. The widespread availability and use of smartphones contribute to the feasibility of electronic bladder diaries in modern society [38]. The automatic processing of electronic reports offers benefits to health care professionals, such as reduced calculation times and errors, which can lead to improved clinical outcomes. Although several studies have demonstrated the feasibility of electronic bladder diaries, it is less clear whether they offer a significant advantage over the paper-based method, owing to conflicting findings [31-36,39]. This may be because of the fact that although an electronic diary eliminates the use of paper, patients are still required to measure the V-voids using a urinary container and manually enter the data into the electronic diary.

### Minze Homeflow a Novel Approach to LUTS Assessment

**Figure 3:**
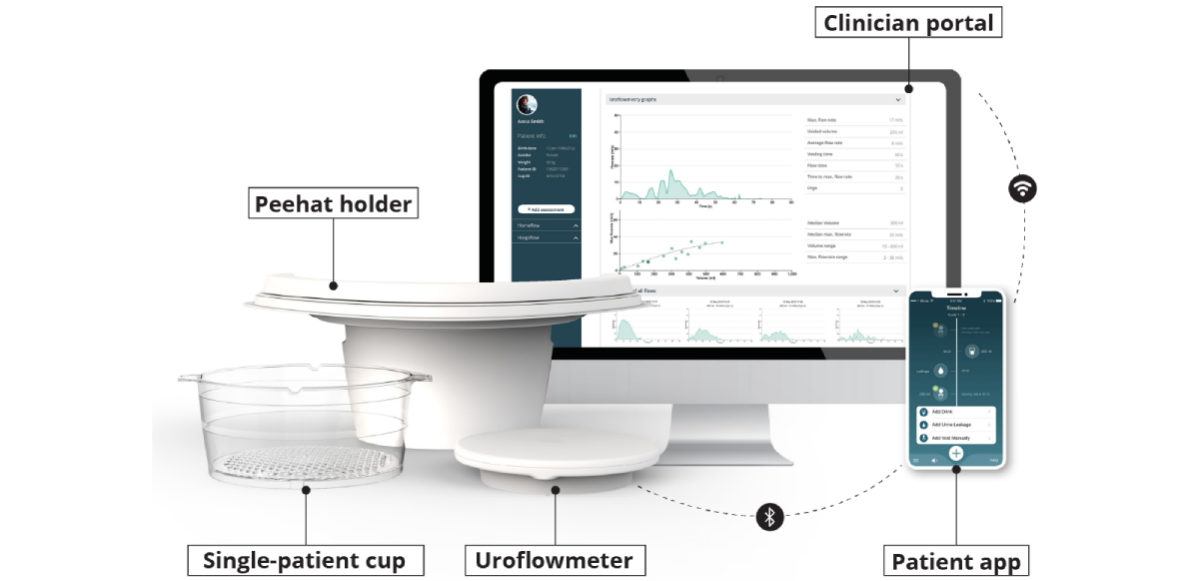
Minze homeflow: Bluetooth-connected gravimetric uroflowmeter with accessories (peehat holder and single-patient cup), patient smartphone app, and cloud-based clinician portal.

Minze homeflow represents an innovative approach for assessing LUTS by combining home uroflowmetry and automated bladder diaries. The system comprises a Bluetooth-connected uroflowmeter, a patient smartphone app, and a cloud-based clinician portal (Figure 3). The uroflowmeter uses gravimetric technology, a well-established and longstanding method, to accurately measure the urinary flow rate. The device has been validated as a class-I measuring medical device, confirming its accuracy in compliance with the Essential Requirements outlined in the European Medical Device Directive. The clinical acceptance of the device was evaluated by the Bristol Urological Institute, which found that the Q-max and V-void measurements met the accuracy criteria outlined in the International Continence Society guidelines [40]. In addition, the device demonstrated a satisfactory response to step changes, well within the International Continence Society recommendations.

**Figure 4:**
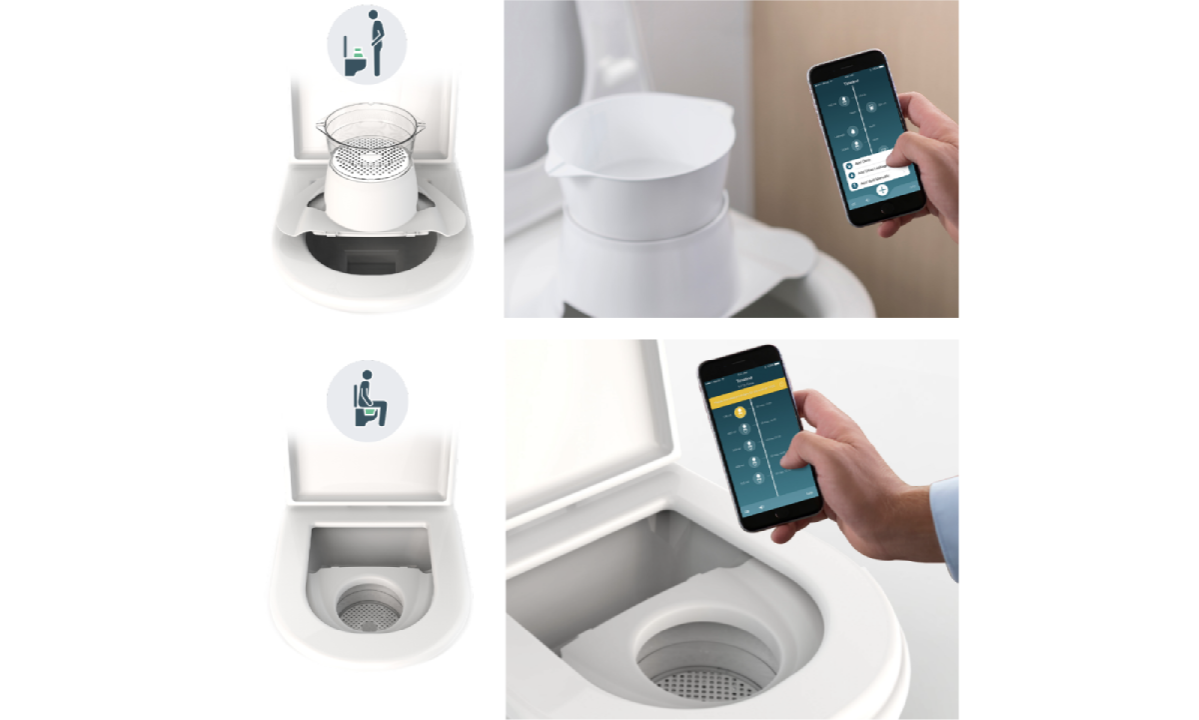
Minze homeflow’s unique design allows for its placement on a standard toilet in either a standing (top) or sitting (bottom) position.

Prior research on home uroflowmetry has been conducted. Golomb et al [41] were among the first to conduct a study on home uroflowmetry in 1992. Following the study by Golomb et al [41], subsequent researchers such as De La Rosette et al [19], Boci et al [42], Porru et al [43], and Heesakkers et al [44] conducted their own studies on home uroflowmetry. Various types of devices were used in these studies: home uroflowmeters that are very similar to the in-clinic model were used by Boci et al [42] and Porru et al [43], handheld uroflowmeters were used by Golomb et al [41] and De La Rosette et al [19], and simple funnel-like devices that provide only an estimate of Q-max were used by Heesakkers et al [44]. However, each of these devices has its own limitations. The in-clinic–like uroflowmeters have the disadvantage of being bulky and expensive, which may limit their widespread use and accessibility. Handheld uroflowmeters, by contrast, are more susceptible to artifacts caused by shaking during use. The simple funnel-like devices offer only a rough estimate of the Q-max and V-void with a large potential error, without generating a complete uroflow curve. In recent years, novel measuring technologies such as sonouroflowmetry have emerged. This technology uses a smartphone to record the sound of the urine stream and convert it into flow rate measurements. However, these technologies often also suffer from accuracy limitations [45]. Furthermore, all the devices used in previous studies were designed exclusively for male patients. By contrast, the Minze homeflow device distinguishes itself with its unique design as it can be placed on a standard toilet in both sitting and standing positions (Figure 4), further enhancing the natural voiding process for both male and female individuals.

In addition to the aforementioned aspects, the Minze homeflow device stands out the most with its mobile health component, making use of the latest technological advancements. The smartphone app offers the additional benefit of including an automated bladder diary with the uroflowmetry measurements. Unlike an electronic bladder diary, the automated diary simplifies the data capture process by automatically collecting voiding data through a connected measuring device. This eliminates the need for manual input or readout of measurements, ensuring convenience and minimizing potential errors. The app also includes prompts and guidance to encourage patient compliance and to ensure proper device use. The direct feedback provided by the system further gives patients confidence and reassurance that the device is functioning appropriately. Moreover, it offers health care professionals the ability to access patient data remotely and in real time, facilitating efficient and timely interventions. This feature sets it apart from the other discussed devices, which require patients to return or send the device by post for physical data retrieval by health care professionals. Such processes can be time-consuming and limit the remote use of the devices. In addition, the cloud-based database stores all data, allowing for digital availability to track changes over time and enabling patients to take ownership of their uroflow and bladder diary data.

In summary, our evaluation has confirmed that the Minze homeflow device is a suitable and reliable option for home uroflowmetry and automated bladder diaries. In this report, we present our first-year experience of managing urological patients at a tertiary urology clinic using the Minze homeflow device.

## Methods

### Overview

We conducted a descriptive retrospective analysis of our first-year experience using the Minze homeflow device, which was performed between July 2019 and July 2020 at a tertiary university hospital (University Hospital Antwerp). Given its compatibility with standard toilets, we used the device in both the clinical (hospiflow) and home (homeflow) settings. As we had no prior experience with homeflow, we selectively offered it to patients for whom we deemed it to be clinically useful or beneficial. The patient received a personal homeflow cup with a near field communication tag, which was linked to the patient in the clinician portal. This eliminated the need for patients to create and remember log-in details, as every measurement was automatically linked to the correct patient through the uroflowmeter’s reading of the near field communication tag. The uroflowmeter is a reusable part that is lent out by the hospital or a homecare shop or sent by post. The patient received an email and a flyer with instructions about using homeflow. The homecare shop provided additional telephone support and assistance. At the end of the assessment, the patients were required to return the uroflowmeter to the hospital or homecare shop for reprocessing. The cloud-based clinician portal was used to access the data remotely and in real time, which included (1) an overview of all homeflow curves; (2) a detailed uroflowmetry graph with key parameters (upon selection); (3) a plot of Q-max versus V-void with median and ranges of Q-max and V-void; and (4) in the case of combined with a bladder diary, automatic calculation of bladder diary parameters. Our objective was 2-fold: first, to provide a description of the reasons for using homeflow and second, to compare the data obtained from homeflow with the data obtained from in-clinic uroflowmetry (hospiflow). Data were obtained from the electronic patient records and the Minze Clinician Portal. To gain insights from the large data set, we used descriptive statistics and Bland-Altman plots of the differences between hospiflow and homeflow. Furthermore, we conducted inferential statistics by using the McNemar test for paired dichotomous data and the nonparametric Wilcoxon signed rank test (owing to nonnormal distribution) to assess differences in Q-max and V-void values within the paired data sets. Statistical analysis was performed using RStudio (version 2023.03.01+446; Posit Software).

### Ethical Considerations

At the University Hospital of Antwerp, robust protocols uphold patient privacy within the institutional framework. GDW and SDW undertook the deidentification of clinical data before subsequent analysis, ensuring the removal of all patient identifiers. Given the focus on analyzing deidentified historical data obtained during routine patient care without any additional patient involvement or compensation, this study is classified as a noninterventional retrospective study. In accordance with Article 3, paragraph 2, of the Belgian law dated May 7, 2004, concerning experiments on human persons (which integrated the European Clinical Trial Directive [2001/20/EC] into the Belgian legal system), this law does not apply to noninterventional retrospective studies. Consequently, the Belgian legal system does not require an ethics review or informed consent for this study.

## Results

### Patient Characteristics and User Statistics

A detailed summary of the patient population and homeflow use is presented in Table 1.

The Minze homeflow device was offered to 166 patients with various urological pathologies following an in-clinic assessment. Of the 166 patients, 91 (54.8%) were children and adolescents with a mean age of 8.8 (SD 3.6; range 2-17) years, whereas 75 (45.2%) were adult patients with a mean age of 42.8 (SD 17; range 18-75) years. Most patients in both groups were male, with 66% (60/91) and 85% (64/75) of pediatric and adult patients, respectively. The mean number of homeflows collected per patient was similar for children and adults, with means of 18.5 (SD 12.9; range 1-97) and 20.5 (SD 14.9; range 1-100), respectively. However, the reasons for recording homeflow measurements were different, with 85% (77/91) of pediatric patients using homeflow for diagnostic purposes and a more equal distribution for adult patients using it in 55% (41/75) of cases for diagnosis and 45% (34/75) of cases for treatment follow-up such as medication or surgery. In addition, 77% (70/91) of pediatric patients used an automated bladder diary combined with the homeflow assessment, whereas only approximately half (39/74, 52%) of the adults did so.

Overall, 15% (14/91) of children and 31% (23/75) of adults were unable to provide a measurement in the hospital, resulting in a total of 129 hospiflow measurements. In comparison, a total of 3214 homeflow measurements were obtained. For further analysis, the homeflow assessments of 5% (5/91) of children and 8% (6/75) of adults were excluded. The reasons for exclusion were insufficient measurements or a high number of artifacts caused by technical or user errors, such as accidental bumping or incorrect placement. However, the occurrence of artifacts remained within an acceptable range, as only 86 (2.68%) out of the 3214 homeflow curves needed to be excluded owing to artifacts.

**Table 1:** Patient population and homeflow use (N=166).

Data characteristics			Pediatric patients (aged <18 years; n=91)		Adult patients (aged ≥18 years; n=75)
**Sex, n (%)**					
	Male	60 (66)		64 (85)	
	Female	31 (43)		11 (15)	
**Age (years)**					
	Range	2-17		18-75	
	Mean (SD)	8.8 (3.6)		42.8 (17)	
	Median (IQR)	8 (6-11)		42 (30-59)	
**Number of homeflows per patient**					
	Range	1-97		1-100	
	Mean (SD)	18.5 (12.9)		20.5 (14.9)	
	Median (IQR)	16 (11-22)		20 (11-26)	
**Homeflow for diagnosis, n (%)**			77 (85)		41 (55)
	Mixed symptoms	8 (9)		21 (28)	
	Urinary incontinence	30 (33)		7 (9)	
	Nocturnal enuresis	20 (22)		1 (1)	
	Recurrent urinary tract infections	10 (11)		5 (7)	
	Urgency or frequency	7 (8)		4 (5)	
	Dysuria	2 (2)		2 (3)	
	Nocturia	0 (0)		1 (1)	
**Homeflow for follow-up, n (%)**			14 (15)		34 (45)
	Urethroplasty	1 (1)		22 (29)	
	Other urological surgery	3 (3)		3 (4)	
	Hypospadia repair	3 (3)		2 (3)	
	Transurethral resection of the prostate	0 (0)		4 (5)	
	Neurogenic bladder monitoring	3 (3)		1 (1)	
	Medication effect	3 (3)		1 (1)	
	SNM^a^ or Botox effect	1 (1)		1 (1)	
With automated bladder diary, n (%)			70 (77)		39 (52)
**Assessments missing or excluded, n (%)**					
	Hospiflow: inability to perform a hospiflow	14 (15)		23 (31)	
	Homeflow: insufficient homeflow measurements or a high number of artifacts	5 (5)		6 (8)	

^a^SNM: sacral neuromodulation.

### Reasons for Homeflow Measurements

On the basis of our 1-year clinical experience with homeflow, we have formulated several reasons for its use, which we have categorized into 2 groups: diagnosis and follow-up.

#### Homeflow for Diagnosis

##### Inability to Perform a Hospiflow

Homeflow can serve as an alternative method when hospiflow fails or proves unfeasible, as may occur in cases of patient stress or incomplete bladder filling. Of the 166 patients in our database, 37 (22.3%) were unable to provide a measurement during in-hospital testing.

##### Young Children

Homeflow can be particularly useful in young children who often face challenges urinating on command and experience discomfort in the in-clinic environment. In addition to providing a more natural environment for the child, homeflow can also save time and reduce inconvenience for parents, who often need to wait for the child to drink enough fluid to fill their bladder before testing. Moreover, following guidelines, at least 2 uroflow curves need to be obtained, further increasing the time spent in the hospital.

##### Verification of Abnormal and Normal Hospiflow Result

Homeflow can also help to confirm or exclude abnormal voiding patterns owing to unreliability caused by the hospital setting or owing to a dysfunction, such as an obstruction, overactive bladder, or pelvic floor dysfunction. Particularly, in cases of staccato or interrupted flow patterns, homeflow helps to distinguish between a persistent voiding dysfunction and a temporary unrelaxed flow caused by stress and distractions associated with the hospital environment. In addition, volume-related abnormal curves such as plateau-shaped curves can be detected using homeflow, which may go unnoticed during in-clinic uroflow measurements when the V-void is too low.

##### Before Invasive Urodynamic Assessment

Homeflow can be a valuable tool in situations preceding invasive urodynamic (pressure-flow) examinations or when conducting such tests is difficult. The ability of homeflow to provide a rich data set offers new insights compared with a single in-clinic measurement. When used in conjunction with a bladder diary, it enables the assessment of voiding habits and uroflowmetry data at varying V-voids, urgency levels, and different times of the day. This rich data set can be beneficial in optimizing urodynamic procedures and interpretation, particularly in assessing factors such as bladder filling volume, the occurrence of urgency sensations, and Q-max.

##### Combined Analysis of Voiding and Drinking Habits

Homeflow, combined with a bladder diary, integrates information regarding fluid intake and urination patterns. The automatically processed data enable convenient identification of potential factors contributing to LUTS, such as small bladder capacity, nocturnal polyuria, or drinking habits. This comprehensive analysis facilitates the delivery of personalized lifestyle advice, including recommendations on hydration levels, establishing consistent voiding habits, and implementing strategies to mitigate the risk of complications related to urinary issues. This approach can be beneficial for patients with various types of LUTS, such as overactive bladder, urinary incontinence, nocturia, nocturnal enuresis, recurrent urinary tract infections, or neurogenic bladder. For instance, we have successfully identified cases where a large intake of fluids in the evening has enabled us to rule out nocturnal polyuria as a potential factor contributing to nocturia or nocturnal enuresis.

#### Homeflow for Follow-Up

##### Assess Effectiveness of Treatment

Homeflow with or without an automated bladder diary can be used to assess the effectiveness of therapy by comparing preintervention data with postintervention data. The automated calculations and uroflowmetry overview in the clinician portal make therapy effectiveness interpretation more straightforward. Homeflow without an automated bladder diary was used for patients undergoing surgical interventions such as transurethral resection of the prostate and urethroplasty. This approach facilitated the follow-up of Q-max in the home setting. Homeflow with an automated bladder diary was used for patients undergoing conservative treatment such as medication, bladder training, or scheduled urination. This approach facilitated the follow-up and management of conditions such as urinary incontinence or an overactive bladder by tracking V-void relative to urgency levels and expected bladder capacity.

##### Postoperative or Recurrent Intervention Home Monitoring

**Figure 5:**
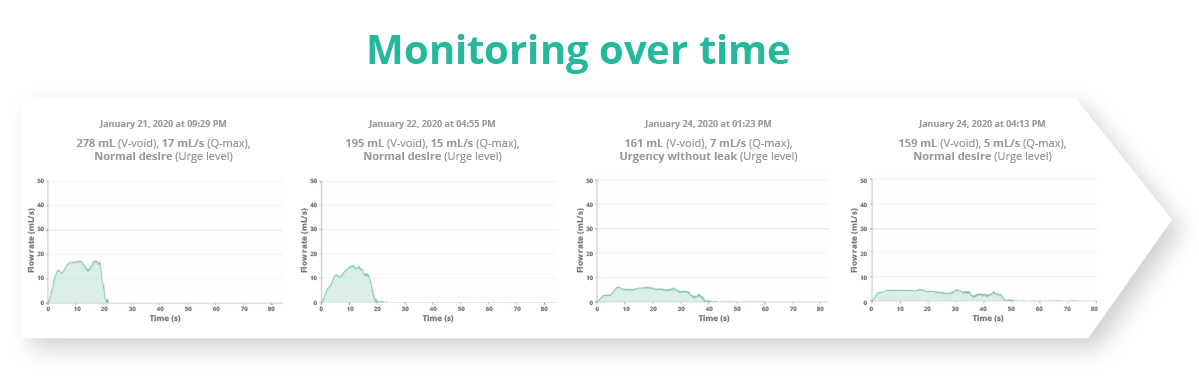
Homeflow for follow-up of a patient receiving urethral dilatation. The need for a new dilation can be detected when the uroflow curve starts to shift toward a plateau-shaped curve again. However, in this particular case, the dilatation had a short effect, as the uroflow curve deteriorated dramatically after only 4 days, leading to the patient’s motivation and agreement to undergo urethroplasty.

The use of homeflow is also appropriate for patients who prefer to be monitored at home. This was the case for a considerable number of our patients, particularly those undergoing postoperative procedures or recurrent interventions. For example, patients who received urethral dilatations were easily followed up at home over a period (Figure 5). This approach facilitated the early detection of the need for a new dilatation and also saved the patients’ time spent on travel and waiting in the clinic for a uroflow test. Similarly, patients with an overactive bladder receiving Botox treatment were also monitored using homeflow. This allowed us to closely monitor their condition and trigger additional Botox injections when required. Textbox 1 includes testimonials from patients who have shared their personal experiences, highlighting the various benefits of using homeflow for remote monitoring.

**Patient testimonials.**

- “Doctor, I would be happy to come and see you in the clinic. However, can I please do my uroflow at home next time? It’s always so stressful for me to do it in the clinic.”
- “Can we arrange my next appointment to be over the phone too? With homeflow, I don’t have to pay for parking, and I don’t have to take a day off work.”
- “Using Minze homeflow was very easy. The app provided me with clear step-by-step guidance throughout the process. Thank you for the successful surgery and for taking good care of me, even from a distance!”

##### Covid-19–Telemonitoring

The COVID-19 pandemic has also prompted the use of homeflow as a tool for telemonitoring. In fact, we used homeflow for telemonitoring in 23.5% (39/166) of our patients to ensure continuity of care during the COVID-19 pandemic, specifically for postsurgery follow-up.

### Analysis of Hospiflow and Homeflow Data

#### Population-Level Comparison

In Table 2, we present the uroflow data, Q-max, and V-void collected from male and female pediatric patients both at the hospital and at home (median values). The first line of the table highlights that the vast majority of these patients were aged ≤12 years. The limited availability of data from older children contributes to establishing a population dataset with increased homogeneity. The data for male and female adult patients are presented in Table 3. The first line of the table indicates that a small majority of male patients were aged ≤50 years, whereas a vast majority of female patients were also in this age group. However, it is important to note that a subset of the patients is not presented in both tables. Table 1 shows that 14 pediatric patients and 23 adult patients were unable to provide a hospiflow measurement, and 5 pediatric patients and 6 adult patients were excluded from the homeflow analysis because of insufficient measurements or a high number of artifacts. The hospiflow data, homeflow data, or both from these patients are not presented.

On a pediatric-population level (Table 2), we observed slightly higher mean median values for both Q-max and V-void at home compared with the mean hospiflow data in male and female pediatric patients. For male patients, we found a mean hospiflow Q-max of 12.6 (SD 6.4; range 3-27) mL/s and a mean homeflow median Q-max of 17.6 (SD 8.3; range 5-36) mL/s. The mean hospiflow V-void for male patients was 119.2 (SD 86.1; range 11-372) mL, whereas the mean homeflow median V-void was 132 (SD 67.3; range 22-282) mL. For female patients, we found a mean hospiflow Q-max of 18.1 (SD 10.7; range 5-43) mL/s and a mean homeflow median Q-max of 21.5 (SD 8.9; range 4-40) mL/s. The mean hospiflow V-void for female patients was 117.6 (SD 106.1; range 21-517) mL, whereas the mean homeflow median V-void was 131.9 (SD 60.9; range 17-244) mL.

For the adult population (Table 3), we also observed a slightly higher mean median value for Q-max at home compared with the mean hospiflow data, but the opposite was observed for V-void. For male adult patients, we observed a mean hospiflow Q-max of 17.3 (SD 9; range 2-39) mL/s compared with a higher mean homeflow median Q-max of 19.5 (SD 8.3; range 8-38) mL/s. The mean hospiflow V-void was 264.3 (SD 187.9; range 14-702) mL compared with a mean homeflow median V-void of 249.3 (SD 126.8; range 12-762) mL. For female adult patients, we observed a mean hospiflow Q-max of 17.7 (SD 11.3; range 6-37) mL/s compared with a higher mean homeflow median Q-max of 23.6 (SD 9.9; range 9-39) mL/s. The mean hospiflow V-void was 235.3 (SD 240.9; range 23-677) mL compared with a mean homeflow median V-void of 193.8 (SD 77.1; range 97-305) mL.

We also examined how many measurements met the recommended minimum V-void criterion for uroflowmetry data, which is 150 mL in adults [15] and at least 50% of age-expected bladder capacity in children [5]. For homeflow assessments, we used the maximum V-void measurement taken at home for this evaluation. In both pediatric and adult patients, we observed that a smaller proportion of hospiflow measurements met the requirement. Specifically, we found that only 37% (19/51) and 35% (9/26) of hospiflow measurements compared with 91% (50/55) and 94% (29/31) of homeflow assessments fulfilled this criterion in male and female pediatric patients, respectively. Similarly, for adult patients, we found 70% (32/46) and 50% (3/11) of hospiflow measurements compared with 93% (54/58) and 100% (11/11) of homeflow assessments met the requirement in male and female adult patients, respectively.

**Table 2:** Pediatric population’s (aged <18 years) hospiflow data (n=77) and homeflow data (n=86).

Data characteristics		Pediatric male patients			Pediatric female patients	
		Hospiflow assessments (n=51)	Homeflow assessments^a^ (n=55)	Hospiflow assessments (n=26)		Homeflow assessments^a^ (n=31)
Aged ≤12 years, n (%)		40 (78)	41 (75)	24 (92)		28 (90)
**Q-max^b^ (mL/s)**						
	Range	3-27	5-36	5-43		4-40
	Mean (SD)	12.6 (6.4)	17.6 (8.3)	18.1 (10.7)		21.5 (8.9)
	Median (IQR)	12 (7.5-15.5)	15 (11-24)	16.5 (11-22)		23 (14-28)
**V-void^c^ (mL)**						
	Range	11-372	22-282	21-517		17-244
	Mean (SD)	119.2 (86.1)	132 (67.3)	117.6 (106.1)		131.9 (60.9)
	Median (IQR)	99 (57-160)	139 (78-182)	90 (54-152)		130 (95-175)
Minimum V-void fulfilled, n (%)		19 (37)	50 (91)	9 (35)		29 (94)

^a^For population uroflow data in homeflow assessments, the median Q-max and median V-void are reported. The maximum V-void of the set of homeflow measurements was evaluated against the minimum V-void threshold.

^b^Q-max: maximum flow rate.

^c^V-void: voided volume.

**Table 3:** Adult population’s (aged ≥18 years) hospiflow data (n=52) and homeflow data (n=69).

Data characteristics		Adult male patients			Adult female patients	
		Hospiflow assessments (n=46)	Homeflow assessments^a^ (n=58)	Hospiflow assessments (n=6)		Homeflow assessments^a^ (n=11)
Aged ≤50 years, n (%)		24 (52)	33 (57)	5 (83)		10 (91)
**Q-max^b^ (mL/s)**						
	Range	2-39	8-38	6-37		9-39
	Mean (SD)	17.3 (9)	19.5 (8.3)	17.7 (11.3)		22.5 (10)
	Median (IQR)	15 (11-24)	18 (14-24)	18 (9-20)		21 (14-29)
**V-void^c^ (mL)**						
	Range	14-702	12-762	23-677		97-305
	Mean (SD)	264.3 (187.9)	249.3 (126.8)	235.3 (240.9)		193.8 (77.1)
	Median (IQR)	195.5 (136-368)	214.5 (182-328)	197.5 (64-273)		157 (137-262)
Minimum V-void fulfilled, n (%)		32 (70)	54 (93)	3 (50)		11 (100)

^a^For population uroflow data in homeflow assessments, the median Q-max and median V-void are reported. The maximum V-void of the set of homeflow measurements was evaluated against the minimum V-void threshold.

^b^Q-max: maximum flow rate.

^c^V-void: voided volume.

#### Individual Patient-Level Comparison

Differences between hospiflow and homeflow data were not only apparent on a population level but also on an individual patient level. These differences are presented in Table 4, which shows discrepancies in Q-max and V-void between the hospiflow and homeflow data. In total, 15 pediatric and 25 adult patients were excluded from the comparison because they did not have both hospiflow and homeflow data. We evaluated whether the uroflow test performed in the hospital fell within the range of homeflow data for Q-max and V-void. For pediatric and adult patients, we found that 29% (22/76) and 48% (24/50) of hospiflow Q-max measurements fell outside the range of homeflow Q-max measurements, respectively. Most patients in both the pediatric and adult groups had a Q-max lower than the minimum homeflow Q-max, with 24% (18/76) and 32% (16/50) of measurements being lower, respectively. For V-void, 33% (25/76) of pediatric patients’ and 44% (22/50) of adult patients’ hospiflow measurements fell outside the range of homeflow V-void measurements.

Subsequently, we investigated the validity of the hospiflow and homeflow assessments based on the recommended minimum V-void criterion for uroflowmetry data at an individual patient level. More than half of the pediatric patients (Table 5) and one-third of the adult patients (Table 6) had hospiflow measurements that did not meet the specified requirement, whereas at least 1 of their homeflow measurements fulfilled the criterion. These observed differences yielded statistically significant results for both the pediatric group (McNemar χ^2^_1_=38.2; *P*<.001) and the adult group (McNemar χ^2^_1_=8.5; *P*=.004).

**Figure 6:**
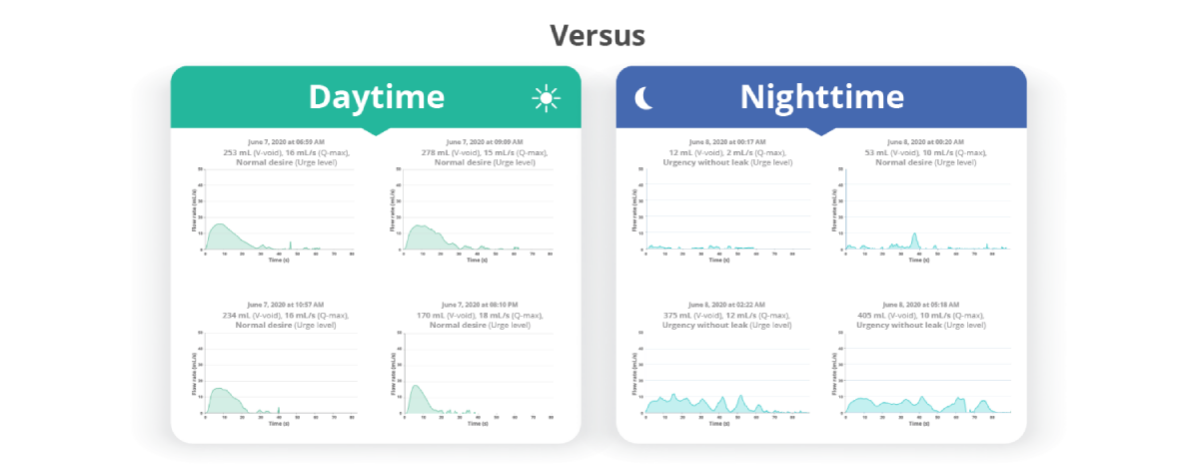
Multiple types of homeflow curves (bell-shaped and staccato) at different times of the day (daytime vs nighttime) observed in a single patient.

**Figure 7:**
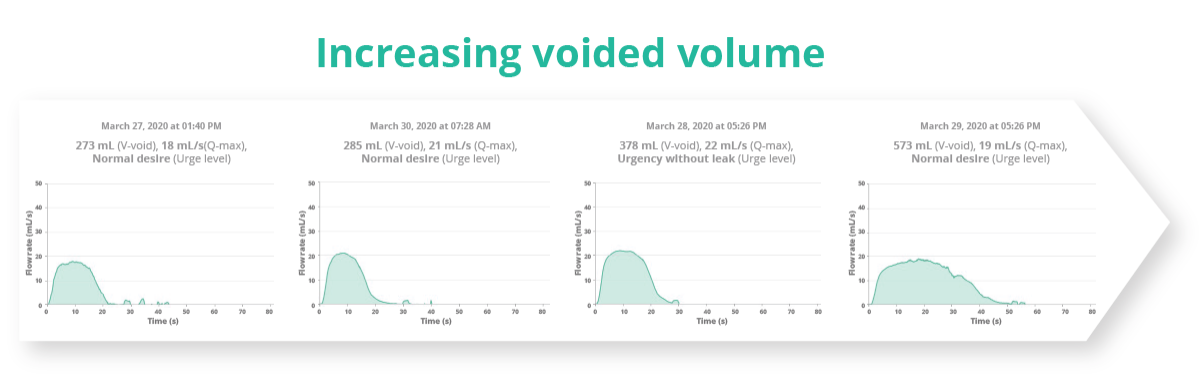
Multiple types of homeflow curves (bell-shaped and plateau) at different voided volumes observed in a single patient.

Further analysis of the shape of the flow curve showed that 36% (27/76) of pediatric patients and 44% (22/50) of adult patients exhibited multiple types of flow curves in their homeflow registrations at different bladder volumes, urge levels, or times of day (Figure 6). This included more plateau-shaped curves at higher volumes (Figure 7).

**Figure 8:**
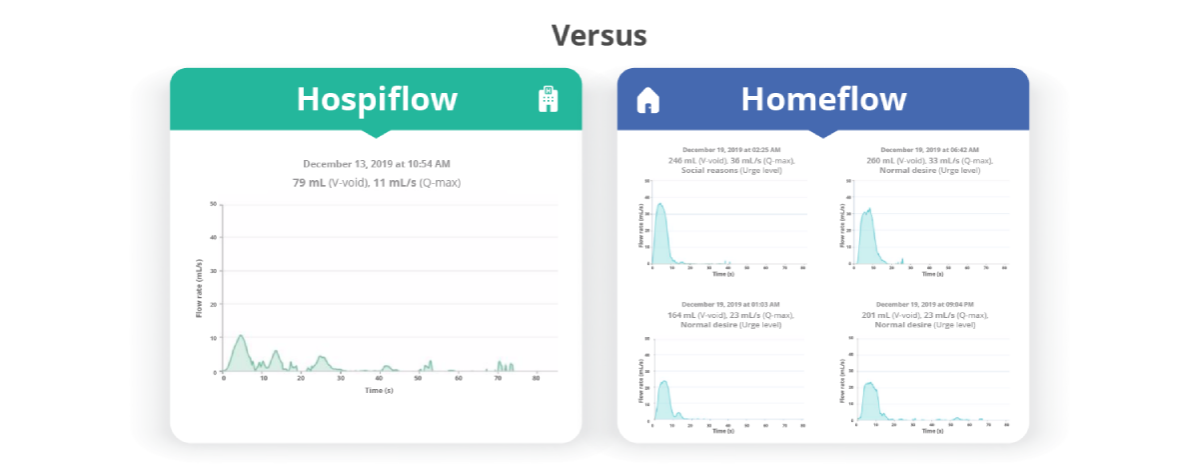
Example of a patient with an abnormal interrupted hospiflow curve and normal bell-shaped homeflow curves.

Moreover, approximately one-fourth of pediatric (Table 7) and adult patients (Table 8) with abnormal flow curves in the clinic were found to have normal bell-shaped flow curves at home (Figure 8). Conversely, some patients were found to have normal flow curves in the clinic but abnormal flow curves at home. In 1 case, this led to the diagnosis of urethral dysplasia following further examination prompted by homeflow. The observed discrepancy between the hospiflow and homeflow curves demonstrated statistical significance in both pediatric patients (McNemar χ^2^_1_=14.5; *P*<.001) and adult patients (McNemar χ^2^_1_=5.1; *P*=.02).

**Figure 9:**
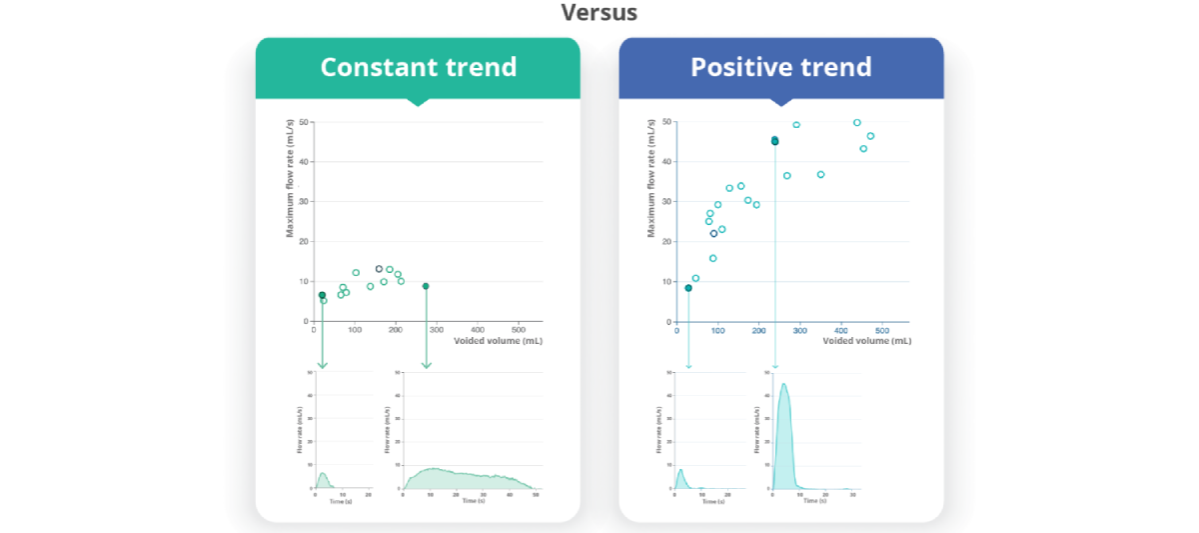
Comparison of maximum flow rate versus voided volume plot in two 9-year-old female pediatric patients with lower urinary tract symptoms, one showing an almost constant correlation (diagnosed with urethral stricture owing to lichen sclerosus) and the other showing a positive correlation (diagnosed with voiding postponement).

Furthermore, homeflow offers the opportunity to explore the association between Q-max and V-void for individual patients across multiple measurements. Our data set revealed diverse trends between these parameters, including positive, negative, and constant trends, which may offer valuable insights for diagnosis. For instance, we observed different Q-max versus V-void plots in two 9-year-old female pediatric patients with LUTS (Figure 9), leading to different dysfunctions. In addition, this parameter can be of interest for follow-up purposes. Despite improvements in the Q-max and normalization of the uroflow curve in some patients, their Q-max versus V-void relationship remained constant.

**Figure 10:**
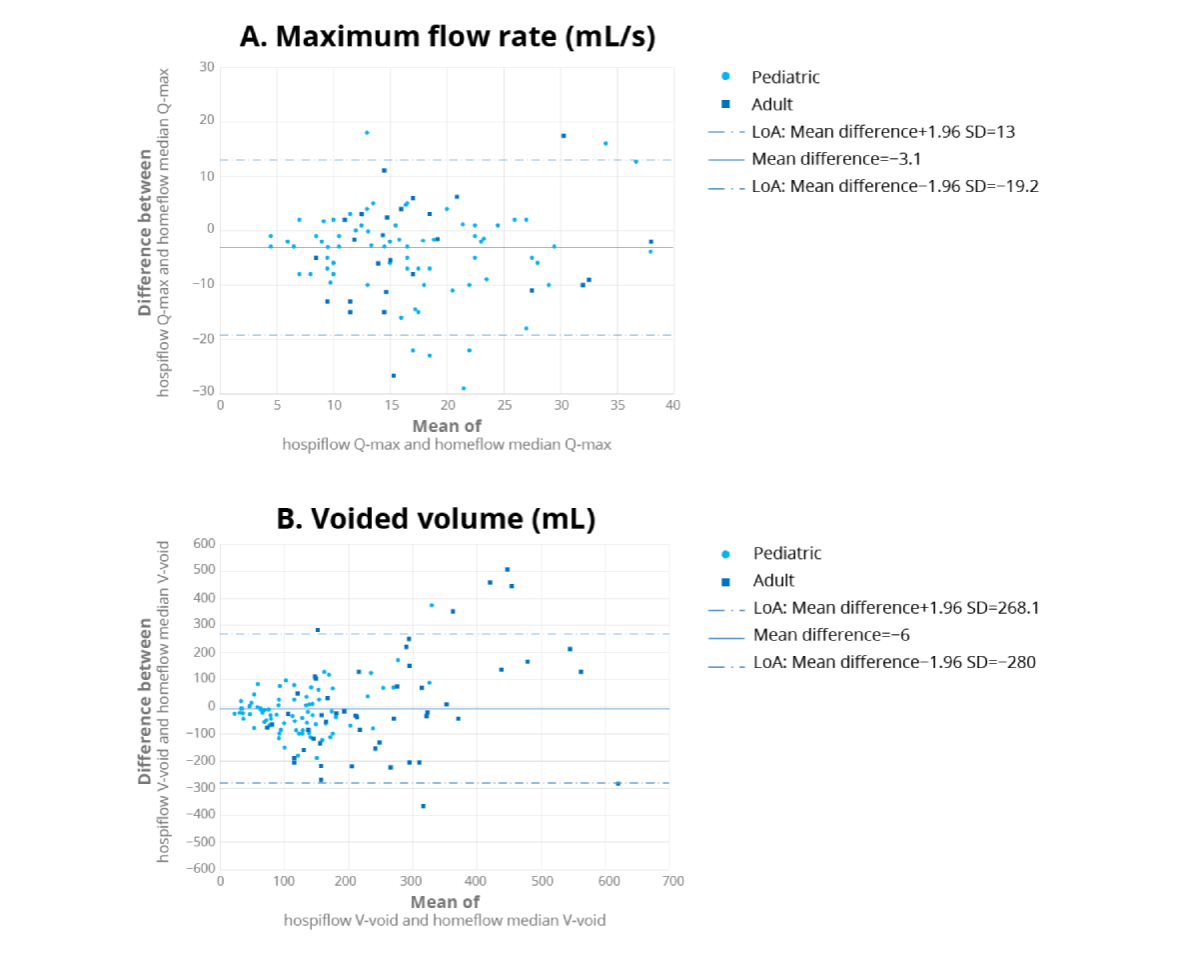
Bland-Altman plot showing the agreement between hospiflow and homeflow for (A) maximum flow rate (Q-max) and (B) voided volume (V-void). Each dot in the plot represents a single patient. The solid line represents the mean difference, and the dashed lines represent the limits of agreement. LoA: limit of agreement.

Finally, we used the Bland-Altman plot to compare hospiflow Q-max and homeflow median Q-max as well as hospiflow V-void and homeflow median V-void (Figure 10). The mean difference between the 2 methods for Q-max was −3.1 mL/s, with an upper limit of agreement (LoA) of 13 mL/s (mean difference+1.96 SD) and a lower LoA of −19.2 mL/s (mean difference−1.96 SD). This difference was found to be statistically significant (Wilcoxon signed rank test: V=2019.5; *P*<.001). For V-void, the mean difference was −6 mL, with an upper LoA of 268.1 mL (mean difference+1.96 SD) and a lower LoA of −280 mL (mean difference−1.96 SD). However, this difference was not found to be statistically significant (Wilcoxon signed rank test: V=3326.5; *P*=.10).

Overall, these results show that the Q-max and V-void measurements obtained with hospiflow and homeflow differ considerably, suggesting that the methods are not equivalent.

In summary, our data show that there are discrepancies between hospiflow and homeflow data. Hospiflow measurements, in many cases, result in artificial curves, which may be attributed to the on-command and unnatural nature of the test. In contrast, homeflow offers a more comprehensive data set, enabling the examination of the Q-max and V-void association for individual patients across multiple measurements. In addition, it allows for the calculation of an averaged, median, and maximum result, providing a potentially more accurate representation of the patient’s voiding function. Thus, relying on a single in-clinic uroflow test may potentially result in missed diagnoses and unnecessary diagnostic or therapeutic procedures.

**Table 4:** Comparison of hospiflow and homeflow data (n=126).

Data characteristics		Pediatric patients (aged <18 years; n=76)	Adult patients (aged ≥18 years; n=50)
**Hospiflow Q-max^a^ to homeflow Q-max range, n (%)**			
	Lower than the minimum value of the range	18 (24)	16 (32)
	Within the range	54 (71)	26 (52)
	Higher than the maximum value of the range	4 (5)	8 (16)
**Hospiflow V-void^b^ to homeflow V-void range, n (%)**			
	Lower than the minimum value of the range	16 (21)	12 (24)
	Within the range	51 (67)	28 (56)
	Higher than the maximum value of the range	9 (12)	10 (20)

^a^Q-max: maximum flow rate.

^b^V-void: voided volume.

**Table 5:** A 2×2 contingency table presenting a comparison of meeting the minimum voided volume (V-void) threshold between hospiflow and homeflow assessment in pediatric patients (aged <18 years; n=76).

Pediatric patients	Homeflow: minimum V-void not fulfilled, n (%)	Homeflow: minimum V-void fulfilled, n (%)	Total, n (%)
Hospiflow: minimum V-void not fulfilled	6 (8)	43 (57)	49 (64)
Hospiflow: minimum V-void fulfilled	1 (1)	26 (34)	27 (36)
Total	7 (9)	69 (91)	76 (100)

**Table 6:** A 2×2 contingency table presenting a comparison of meeting the minimum voided volume (V-void) threshold between hospiflow and homeflow assessment in adult patients (aged ≥18 years; n=50).

Adult patients	Homeflow: minimum V-void not fulfilled, n (%)	Homeflow: minimum V-void fulfilled, n (%)	Total, n (%)
Hospiflow: minimum V-void not fulfilled	1 (2)	15 (30)	16 (32)
Hospiflow: minimum V-void fulfilled	2 (4)	32 (64)	34 (68)
Total	3 (6)	47 (94)	50 (100)

**Table 7:** A 2×2 contingency table presenting a comparison of hospiflow and homeflow curves in pediatric patients (aged <18 years; n=76).

Pediatric patients	Homeflow: abnormal, n (%)	Homeflow: normal, n (%)	Total, n (%)
Hospiflow: abnormal	7 (9)	19 (25)	26 (34)
Hospiflow: normal	1 (1)	49 (64)	50 (66)
Total	8 (11)	68 (89)	76 (100)

**Table 8:** A 2×2 contingency table presenting a comparison of hospiflow and homeflow curves in adult patients (aged ≥18 years; n=50).

Adult patients	Homeflow: abnormal, n (%)	Homeflow; normal, n (%)	Total, n (%)
Hospiflow: abnormal	15 (30)	13 (26)	28 (56)
Hospiflow: normal	3 (6)	19 (38)	22 (44)
Total	18 (36)	32 (64)	50 (100)

## Discussion

### Principal Findings

Our study demonstrated the usefulness of homeflow in urology, with the Minze homeflow device proving to be a valuable tool in various patient categories. Our data show that Minze homeflow is suitable for use among patients of all sexes and age groups, as our patient population included both male and female individuals aged between 2 and 75 years. We identified several reasons for the use of homeflow within 2 main groups: diagnosis and follow-up. It is valuable for diagnosis when hospiflow is unreliable, for confirming or excluding abnormal hospiflow results, and for providing insights before invasive urodynamic examinations. The integration of an automated bladder diary enables the combined analysis of voiding and drinking habits, facilitating the identification of various factors that may contribute to LUTS, including a small bladder capacity, evening fluid intake, and other relevant parameters. Our comparison of hospiflow and homeflow data revealed notable discrepancies in Q-max, V-void, and uroflow curve shape. Our findings suggest that hospiflow fails to provide an accurate representation of a patient’s natural voiding in a considerable number of cases, with approximately one-fourth of patients displaying abnormal flow curves during in-clinic assessments demonstrating normal bell-shaped flow curves at home. In contrast, homeflow leads to more representative voiding results, with a significantly higher percentage of assessments meeting the recommended minimum V-void for proper uroflowmetry assessment. In addition, homeflow provides us with a Q-max and V-void association and an average or median result, which we found to differ significantly from the single in-clinic measurement for Q-max. These results suggest that homeflow is more reliable. This higher level of reliability makes it a valuable tool not only for diagnosis but also for follow-up assessments to assess treatment effectiveness, for postoperative and recurrent intervention home monitoring, and for telemonitoring during the COVID-19 pandemic.

### Comparison With Prior Work

As stated in the Introduction section, Golomb et al [41] were among the first researchers to conduct studies on home uroflowmetry in 1992, and their findings align with those of our study in many aspects. Their findings highlighted circadian changes and variability between consecutive flows using home uroflowmetry. They emphasized that making clinical decisions based on a single-flow measurement is questionable owing to this variability. Their research also demonstrated that home uroflowmetry, in contrast to in-clinic uroflowmetry, offers valuable insights into the reproducibility of abnormal findings. This is particularly relevant for Q-max measurements, which can be influenced by factors such as initial bladder volume. Bray et al [46] supported this finding in their literature review, demonstrating that home uroflowmetry improved the diagnostic accuracy by averaging multiple measurements of Q-max. Following the study by Golomb et al [41], subsequent researchers such as De La Rosette et al [19], Boci et al [42], Porru et al [43], and Heesakkers et al [44] further emphasized the benefits of home uroflowmetry. Their studies demonstrated that home uroflowmetry provides more detailed information compared with traditional single-void uroflowmetry.

When comparing our findings with those of the aforementioned studies [19,42-44], we observed similar results regarding the V-void, as both our study and the previous studies reported differences between home and in-clinic measurements. However, in contrast to our finding, concerning Q-max, previous studies found a close relationship between measurements from both methods. Heesakkers et al [44] even reported no differences in both Q-max and V-void. A potential explanation for the differences in the Q-max findings between our study and the previous studies can be attributed to both the patient demographics covered by the study and the type of homeflow device used. All previous home uroflowmetry studies focused exclusively on adult male patients with bladder outlet obstruction, often related to prostate enlargement. As discussed in detail in the Introduction section, the devices used in the aforementioned studies were designed exclusively for male patients. In addition, each of these devices had its own set of limitations, encompassing factors such as convenience, price, accuracy, and the lack of remote connectivity. In particular, the results obtained from the simple funnel-like device used by Heesakkers et al [44] need to be nuanced because it provides only a rough estimate of Q-max and V-void with a larger potential error. In contrast, the Minze homeflow device has an inclusive design, making it suitable for use by individuals of all ages and sexes. This enabled us to include a diverse patient group, encompassing individuals of various ages, sexes, and presenting with different types of LUTS. This broader applicability and inclusivity of patient demographics may contribute to the observed discrepancies in the Q-max findings.

Finally, it is important to note that patients have consistently shown a preference for homeflow over clinic-based uroflowmetry. This preference was observed in the studies conducted by Boci et al [42] and Heesakkers et al [44], and it was further evident in our study through patient quotes expressing satisfaction and a clear preference for homeflow. Patients cited reduced stress and time-saving benefits as key factors in their preference for homeflow. These findings are in line with the research by Winkelman et al [47], who reported a median travel time savings of 2.25 hours through remote video visits. However, a limitation of remote consultations is the inability to perform measurements. This is where homeflow becomes a valuable addition to telemedicine consultations, as it enables remote and real-time monitoring of physiological voiding parameters. Overall, homeflow offers a patient-centered approach to uroflowmetry, addressing the preferences and needs of patients while providing accurate and reliable data for clinical assessment.

### Benefits and Potential Improvements of Minze Homeflow

We evaluated the use of the Minze homeflow device as a novel approach for managing urological patients. Our experience revealed that the device was easy to set up and enabled automatic data collection with minimal effort required from both patients and physicians. In addition, its mobility and portability allowed patients to perform uroflowmetry in their preferred setting, be it at home, work, or school. Contrary to potential biases or assumptions about the older population’s ability to use a smartphone-connected device such as the Minze homeflow, our findings demonstrated that age and sex did not pose any significant challenges in using the homeflow device. For patients, the main advantages of homeflow included increased involvement in treatment, the ability to perform uroflowmetry in the comfort of their home, and a reduction in travel time and time off work or school. For physicians, the device provided more comprehensive and reliable data for diagnosis and follow-up, increased practice efficiency, and reduced waiting lines, particularly given the challenges posed by the COVID-19 pandemic.

The limitations of homeflow include the lack of measurement of postvoid residual volume using ultrasound, which requires the urologist to assess each case and determine whether to perform the test in the office. In addition, we observed some artifacts in the homeflow uroflowmetry data, which may have been because of patient misuse, device sensitivity, or accidental bumps. These artifacts can be manually discarded; however, the process can be time-consuming in case of multiple measurements. However, we observed a lower incidence of artifacts (86/3214, 2.68% of homeflow curves excluded) compared with previous research on home uroflowmeters. Boci et al [42] reported 11.6% of flows being affected by artifacts, and De La Rosette et al [19] found possible artifacts in 21% of the flows with their handheld solution. It is important to acknowledge that all uroflowmetry studies are subject to numerous artifacts. In a clinical setting, these artifacts often stem from environmental and psychological factors such as lack of privacy, stress, and inhibitions related to urinating on command. At home, we can assume that these factors are limited, as patients can void in a more relaxed and natural environment. However, artifacts during home uroflowmetry may still arise from use errors owing to incorrect patient instructions or incorrect use by the patient. Therefore, patients must be carefully instructed on how to use the device, and the design needs to be robust, mobile, simple to use, and accurate to ensure reliable results. In this regard, the Minze homeflow device appears to effectively address these requirements and even surpasses other devices in certain aspects, making it a highly suitable choice for home uroflowmetry. However, minimizing these artifacts or using a smart algorithm for their automatic detection and removal are suggestions for improvement.

Finally, we would like to address some challenges associated with the Minze homeflow device. Owing to the use of a reusable uroflowmeter and single-patient accessories, logistical challenges may arise when receiving and returning the reusable parts. In our case, there were multiple options for the patient to obtain the device, either through the hospital, the homecare shop, or via the post office delivered to the front door in an anonymous package. This method of delivery was often preferred because it aligns with the current trend of web-based ordering and further reduces travel time. Despite the already relatively low cost of the homeflow method, delivering the device through this method results in additional fuel and time savings, further reducing the overall cost for the patient. With regard to support, there is a need for a service that provides additional support when needed, both for the patient (eg, during homeflow device setup) and the physician (eg, when using the cloud-based clinician portal). Overcoming support and logistical challenges can be achieved by implementing effective systems within a country or hospital. However, a significant obstacle lies in the absence of reimbursement for home uroflowmetry in contrast to in-clinic uroflowmetry. This discrepancy is surprising considering that our study, along with previous research, has demonstrated the feasibility, enhanced reliability, patient preference, and potential time and cost savings associated with home uroflowmetry. As the field of digital health continues to advance, the reimbursement pathways for digital health solutions are evolving. Reimbursing home uroflowmetry could be a game changer by eliminating one of its main challenges.

### Future Work

Our study findings, along with those of other studies, raise questions about the reliability of a single in-clinic measurement. Homeflow provides more comprehensive data with multiple measurements taken at different bladder volumes, urge levels, and times of the day. This allows the calculation of an averaged or median Q-max, which may be a more accurate parameter for diagnosing and monitoring patients. Current uroflowmetry population nomograms are based on single in-clinic measurements of individuals. However, the inclusion of multiple homeflow measurements for an individual can enhance these nomograms. This approach would take into account the natural variability in voiding patterns, including the individual Q-max versus V-void relationship. Our homeflow data set revealed that this plot can exhibit positive, negative, or constant trends. By considering these individual patterns, more accurate and personalized nomograms can be developed to better assess and understand an individual’s voiding function. Further research is needed to explore the clinical significance of our findings and to evaluate the potential clinical implications of an individual Q-max versus V-void relationship and nomograms. Finally, in terms of future development, the Minze mobile health ecosystem serves as a foundation for future advancements, including the use of smart algorithms and artificial intelligence for diagnostic classifications, lifestyle recommendations, treatment support, and long-term care for patients with chronic conditions.

### Strengths and Limitations

The strengths of this study lie in its use of a large retrospective database consisting of 166 urological patients, covering a broad range of patient categories, including both pediatric and adult populations as well as male and female individuals. This enabled a comprehensive analysis of the reasons for using homeflow. The analysis is further supported by the large amount of uroflowmetry data, including 3214 homeflows and 129 hospiflows. However, it is important to note that this study was conducted in a Belgian tertiary university hospital, which may differ in terms of patient population and resources from those in peripheral hospitals, other types of specialists, and other health care systems. In addition, the comparison between hospiflow and homeflow is indicative rather than exact, as some patients were under treatment, potentially contributing to some of the observed differences. As this was a retrospective noncontrolled study, randomized controlled trials are necessary to better evaluate the effectiveness of the Minze homeflow device and the differences between homeflow and hospiflow data. Thus, considering these limitations, it is important to interpret the statistical test results with caution.

### Conclusions

In conclusion, our first-year experience with Minze homeflow in our tertiary university hospital was positive, demonstrating its feasibility and usefulness for various patient categories. We identified several reasons for using homeflow for diagnosis and follow-up. For diagnosis, homeflow can be a valuable tool, especially in cases where hospiflow is unreliable or fails, particularly in young children. It can confirm or exclude abnormal hospiflow results and provide valuable insights before invasive urodynamic examinations. In addition, when combined with an automated bladder diary, it enables the combined analysis of voiding and drinking habits, facilitating the identification of various factors that may contribute to LUTS. For follow-up, homeflow can be used to assess the effectiveness of treatment and monitor postoperative or recurrent interventions at home. Homeflow was particularly useful for telemonitoring during the COVID-19 pandemic, ensuring continuous and remote care. Furthermore, we found significant differences between hospiflow and homeflow in terms of Q-max measurements and uroflow curve shape. Our findings suggest that homeflow provides more reliable and comprehensive voiding data compared with hospiflow. The examination of an individual’s Q-max versus V-void plot and the calculation of an averaged or median Q-max enhanced the diagnostic accuracy of uroflowmetry as compared with a single in-clinic measurement. The device was found to be user-friendly, enabling remote data collection with minimal effort required from patients and physicians. As a result, we decided to continue using Minze homeflow in our clinic.

## Data Availability

The data sets generated and analyzed during this study are not publicly available owing to patient privacy and confidentiality concerns but are available from the corresponding author upon reasonable request.

## Abbreviations

LoA: limit of agreement
LUTS: lower urinary tract symptoms
Q-max: maximum flow rate
V-void: voided volume
